# Stentless Intravascular Lithotripsy (IVL) for the Paravisceral Coral Reef Aorta: A Case Report

**DOI:** 10.7759/cureus.90675

**Published:** 2025-08-21

**Authors:** Raoul Borioni, Alessandra Rinaldi Garofalo, Maria Chiara Tesori, Alessia Salerno, Patrizia Alba Gentile

**Affiliations:** 1 Vascular Surgery, Aurelia Hospital, Rome, ITA

**Keywords:** coral reef aorta, intravascular lithotripsy, lower limb ischemia, percutaneous transluminal balloon angioplasty, peripheral arterial diseases

## Abstract

Coral reef aorta (CRA) is an obstructive disease characterized by heavy calcification of the paravisceral abdominal aorta, leading to impairment of visceral perfusion and exercise-limiting lower limb claudication. CRA is traditionally treated with open surgery through a retroperitoneal approach. In recent years, percutaneous angioplasty of the abdominal aorta, combined with open cell stenting or more complex stent-graft procedures, has been proposed as a less invasive alternative to open surgery, but the presence of heavy calcifications can be a relevant issue. The availability of the intravascular lithotripsy (IVL) technology allows for a more effective endovascular treatment of the aortic stenosis associated with significant calcifications.

We report a case of a 55-year-old female patient presenting with bilateral exercise-limiting claudication and no palpable femoral pulses, secondary to paravisceral CRA. An endovascular treatment was performed using an IVL without stenting through a percutaneous right femoral artery access. Following the procedure, a significant improvement in the arterial pressure values was gained at the level of the distal aorta as a result of plaque remodeling. After 40 days, the patient has no recurrence of claudication. Duplex ultrasound confirms triphasic arterial waveforms at the level of the bilateral femoral artery.

Although open surgery continues to be an effective procedure for CRA, IVL can be regarded as an attractive endovascular option when a less invasive procedure is required. A significant hemodynamic improvement can be achieved independently of stent placement. Further clinical experience is required to define the long-term role of this procedure.

## Introduction

Coral reef aorta (CRA) is a low-frequency obstructive disease (0.6%) characterized by heavy calcification of the abdominal aorta, particularly in the visceral and juxtarenal tracts. The rock-hard calcifications project into the aortic lumen with potential involvement of the celiac axis, superior mesenteric, and renal arteries, leading to chronic visceral ischemia and nephrovascular hypertension. As a result of the decreased infrarenal aortic pressure, exercise-limiting lower limb claudication is the most common clinical presentation of CRA [1]. 

CRA is traditionally treated with open surgery through an extraperitoneal approach as originally proposed by Ricotta and Williams [2, 3], eventually combined with aortofemoral bypass grafting [4]. Recent single-center studies [5, 6] confirm that open surgery continues to be an effective procedure for CRA, showing low operative mortality (0% to 2.6%), with a not-negligible short-term morbidity (7% to 29%) and risk of recurrence (up to 15%).

In recent years, endovascular treatment by the use of open-cell stents or covered stent-grafts [7, 8] is emerging as a less invasive procedure for CRA, although the presence of extensive calcification may adversely affect the outcome due to the lower stenting effectiveness. Based on literature experiences in patients with coronary and peripheral arterial disease, an innovative approach using intravascular lithotripsy (IVL) has been proposed for the treatment of CRA [9-15]. This novel technology induces multiplane and longitudinal calcification fractures, delivering acoustic shockwaves to the arterial wall through an angioplasty balloon. As a consequence, an increased vessel compliance associated with luminal gain can be achieved [11, 12] with a low need for stenting [13-15].

In the recent literature, a stentless procedure using IVL for paravisceral CRA has been reported rarely [16-18]. In this paper, we report a case of a 54-year-old patient presenting with bilateral exercise-limiting claudication due to severe paravisceral CRA, treated by IVL with no stent placement, confirming the feasibility of a stentless procedure with significant short-term benefits.

## Case presentation

**Figure 1:**
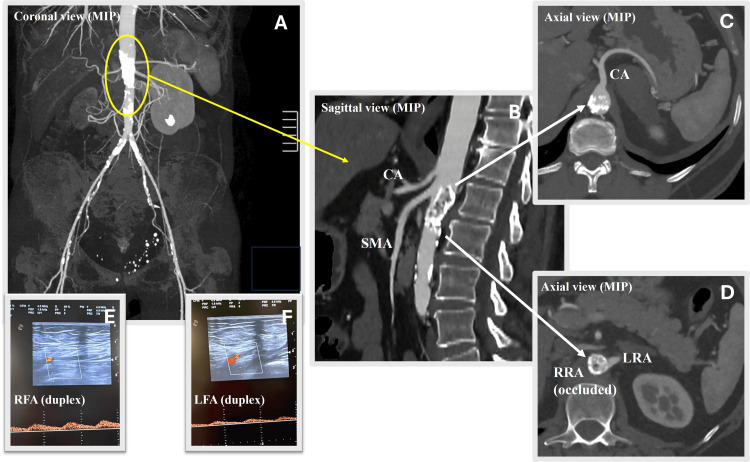
CT angiography (MIP) showing the coral reef aorta (A), with partial involvement of the superior mesenteric artery (B), celiac axis (C), and renal vessels (D). Decreasing aortoiliac perfusion is evident by the arterial monophasic waveform resulting from duplex scanning (E, F). MIP: maximum intensity projection; CA: celiac axis; SMA: superior mesenteric artery; LRA: left renal artery; RRA: right renal artery; RFA: right femoral artery; LFA: left femoral artery

A 55-year-old female patient with a medical history of hypertension, dyslipidemia, obesity, and tobacco use presented with bilateral exercise-limiting claudication (walking distance <100 meters) and no palpable femoral pulses. The ankle-brachial index (ABI) was not evaluable. Vascular duplex ultrasound revealed an arterial monophasic waveform at the level of the bilateral common femoral artery. An abdominal CT scan showed a marked narrowing of the paravisceral aorta (zones 6, 7, and 8, according to the Ishimaru classification) due to heavy calcification, associated with obstruction of the right renal artery (Figure 1). Preoperative lab studies revealed a mild impairment of renal function (serum creatinine 1.41 mg/dl, with a glomerular filtration rate (GFR) of 60 ml/min). Coronary angiography documented an insignificant disease with a normal left ventricular ejection fraction (LVEF) of 57%.

Although a visceral aortic endarterectomy through a left-sided lumbotomy could be the first-line option, an endovascular treatment was preferred, considering the surgical risk and the patient’s preference. Despite the presence of heavy calcifications, the availability of the Shockwave IVL L6 catheter (Shockwave Medical Inc., Santa Clara, CA), with the appropriate size for the treatment of the aorta, suggested the possibility of dealing with no implantable device and performing a stentless procedure.

**Figure 2:**
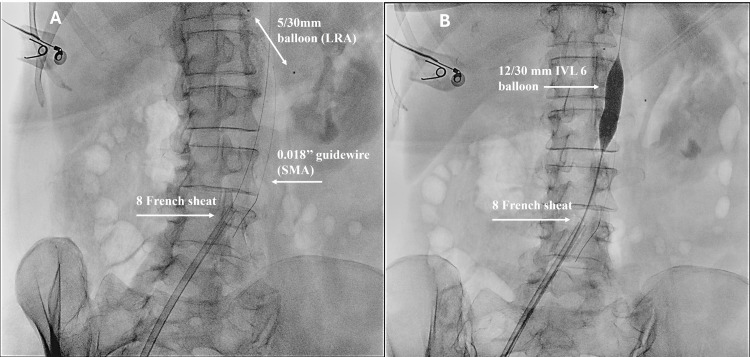
Endovascular setting to perform angioplasty of paravisceral aorta: (A) 0.018’’ guidewire and 5/30 mm balloon catheter from the left brachial sheath to respectively protect the SMA and the LRA; (B) Shockwave IVL L6 catheter (12 mm x 30 mm) advanced through the aortic stenosis on a 0.018’’ guidewire from the right femoral artery. LRA: left renal artery; SMA: superior mesenteric artery; IVL: intravascular lithotripsy

The patient underwent a percutaneous endovascular procedure under general anesthesia in May 2025. A 6F sheath (Shoocin Radial Introducer, Lepu Medical Technology Ltd., Beijing, China) was placed through the left brachial artery, and an 8F 60 cm-long sheath (Durasheat, CMI GmbH, Dresden, Germany) was placed through the right femoral artery. A 0.018’’ Asahi Halberd 18 guidewire (Kardia srl, Asahi Intecc Group, Milan, Italy) was advanced through the aortic stenosis to support the Shockwave IVL L6 Catheter (12 mm x 30 mm). A second 0.018 guidewire and a 5/30 mm balloon catheter were advanced through the left brachial sheath to protect the superior mesenteric artery and the left renal artery, respectively (Figure 2).

**Figure 3:**
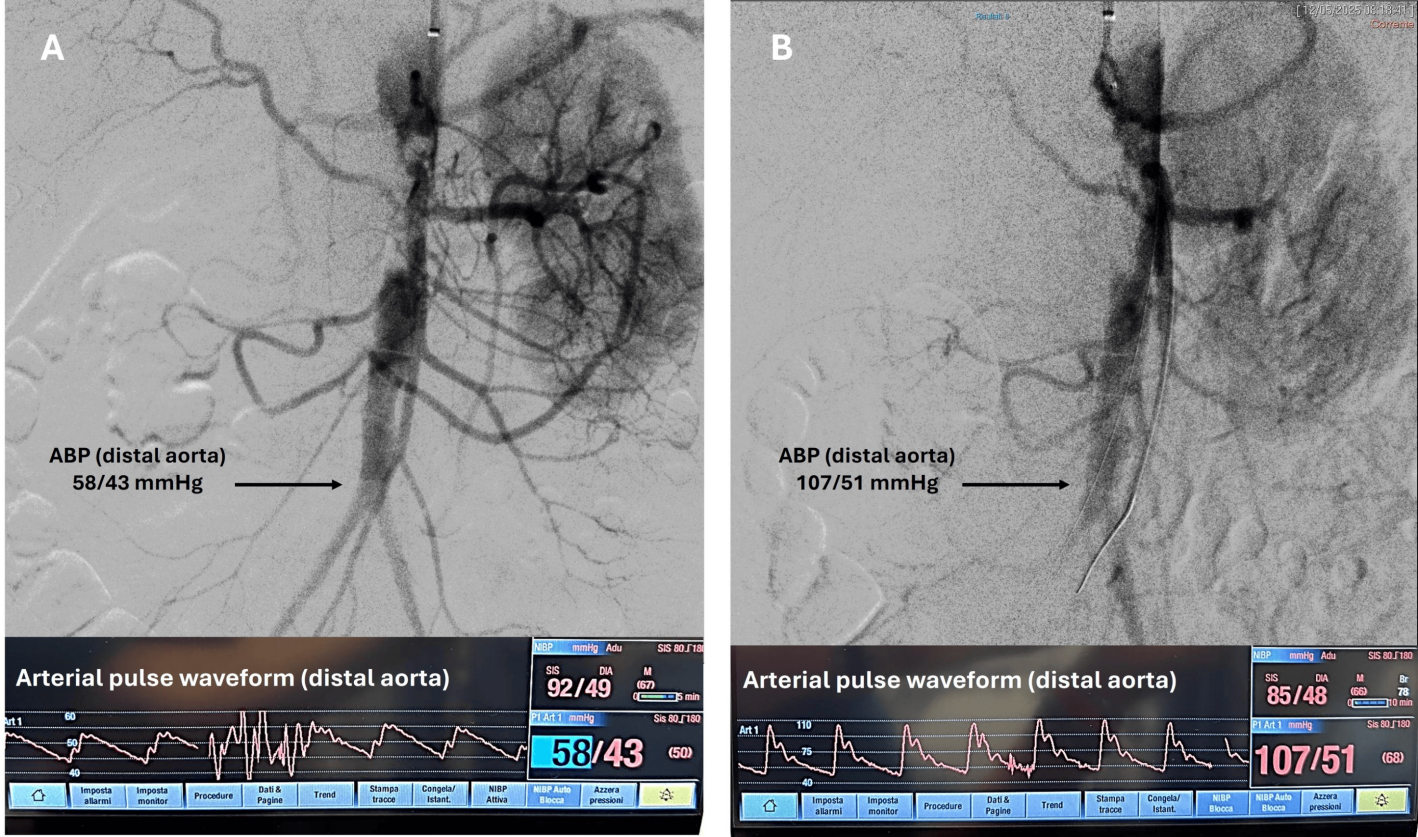
Intraoperative angiography and hemodynamic data: (A) pre-procedural angiogram and hemodynamic data showing low pressure at the level of the infrarenal aorta (58/43 mmHg), compared to the systemic pressure values (92/49 mmHg), with an abnormal arterial pulse waveform at the level of the distal aorta; (B) post-procedural angiogram and hemodynamic data showing a significant improvement of the arterial pressure values (107/51 mmHg) and pulse waveform at the level of the distal aorta, secondary to plaque remodeling, with patency of the visceral vessels. ABP: arterial blood pressure

IVL was performed (180 pulses) in the abdominal aorta using a 12/30 mm Shockwave L6 balloon (Shockwave Medical Inc.) inflated to 2 atm for the first treatment cycle and 4 atm for the second treatment cycle. No postdilation was performed. The post-procedural angiography showed a plaque remolding, with patency of the visceral vessels, associated with a significant improvement of the arterial pressure values and pulse waveform at the level of the distal aorta, even though the angiogram appeared to be suboptimal (Figure 3).

**Figure 4:**
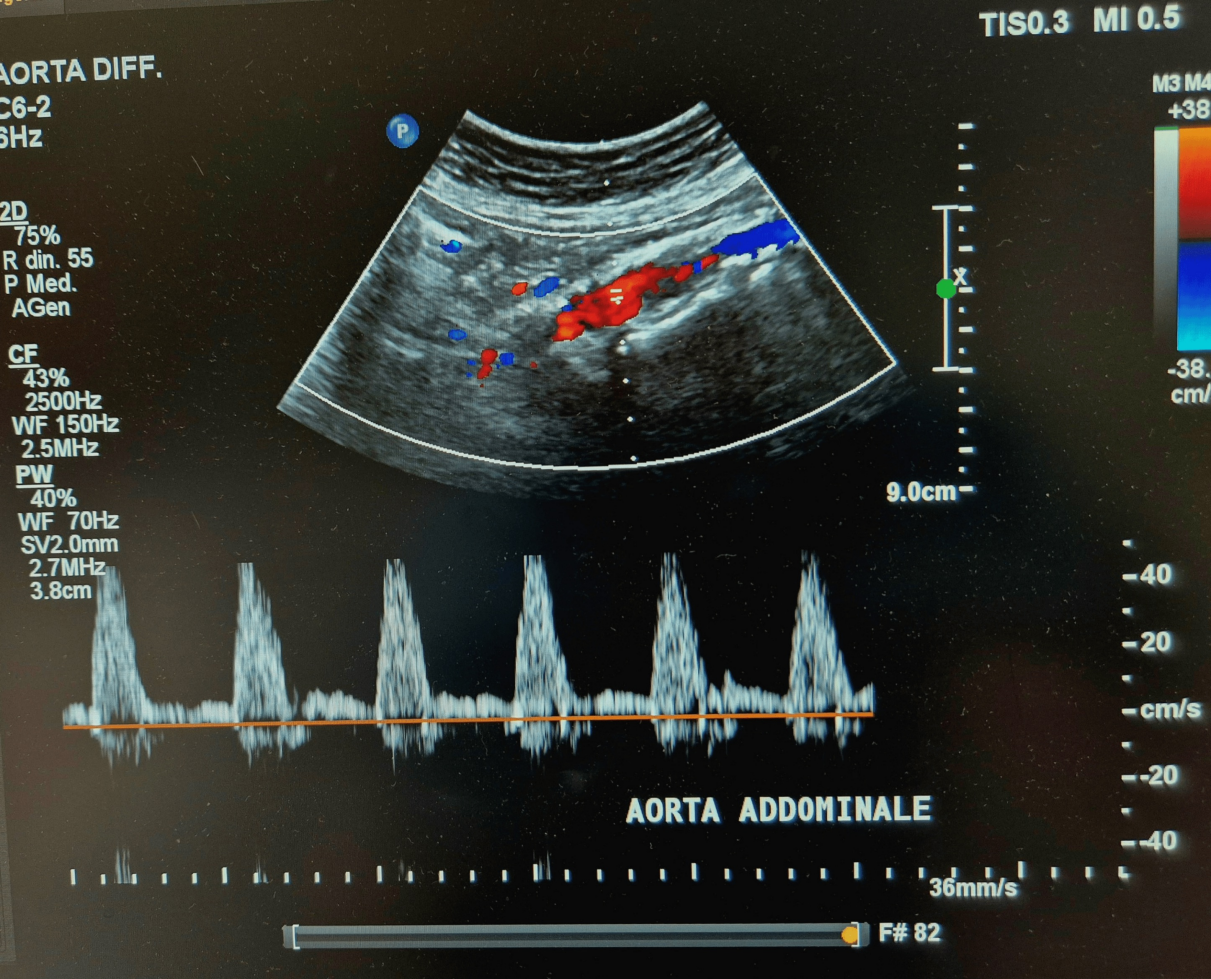
Intraoperative duplex ultrasound evaluation of the infrarenal abdominal aorta showing a triphasic arterial waveform.

Intraoperative duplex ultrasound showed a triphasic waveform at the level of the abdominal aorta, confirming the hemodynamic result (Figure 4).

The right femoral access was closed by means of a single suture-mediated device (Perclose ProGlide, Abbott Inc., Chicago, IL), and the left brachial access was managed by manual compression. The procedural duration was 105 minutes (fluoroscopy time 25 minutes, cumulative Kerma of 648.3 mGy, cumulative dose area product (DAP) 195.12 Gy/cm²), using 130 ml of contrast medium. The postoperative period was uneventful.

**Figure 5:**
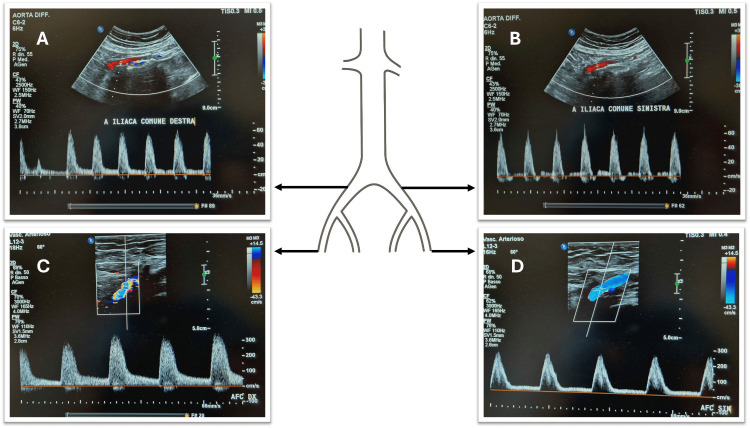
Duplex ultrasound evaluation performed 40 days after the IVL procedure, confirming the hemodynamic results: (A, B) right and left common iliac arteries; (C, D) right and left common femoral arteries. IVL: intravascular lithotripsy

The patient was discharged 48 hours after the intervention with palpable femoral pulses and restored ABI (right leg 0.9, left leg 0.8). Her daily medication included rosuvastatin 10 mg, ezetimibe 10 mg, ASA 100 mg, clopidogrel 75 mg, and telmisartan 40 mg. At 40-day follow-up, she had no recurrence of severe claudication, with a walking distance of 800 meters. Duplex ultrasound confirmed promising results, showing triphasic arterial waveforms (Figure 5). A postoperative CT scan has been scheduled two months later.

## Discussion

CRA is defined as a low-frequency obstructive disease (0.6%) characterized by heavy calcification of the visceral aorta, with significant aortic stenosis leading to lower limb claudication, chronic mesenteric ischemia, or nephrovascular hypertension [1]. The traditional treatment of CRA is open surgery, consisting of endarterectomy of the upper abdominal aorta through an extraperitoneal approach, eventually combined with aortofemoral bypass grafting, as originally proposed by Ricotta and Williams [2-4]. Recent single-center studies [5, 6] confirm that open surgery continues to be an effective procedure for CRA, showing low operative mortality (0% to 2.6%), with a not-negligible short-term morbidity (7% to 29%) and risk of recurrence (up to 15%).

Endovascular treatment, using open-cell stents or more complex stent-graft procedures, is emerging as a less invasive alternative for the cure of CRA [7, 8], particularly since the IVL procedure is available [9-11]. This technology induces multiplane and longitudinal calcification fractures, resulting in an increased vessel compliance with a luminal gain [11, 12]. Chag and Thakre [16] reported the first case of a stentless CRA treatment using a 6/60 mm Shockwave M5 balloon catheter coupled with a 9/30 mm plain balloon catheter to achieve an effective aortic diameter. Two similar cases have been recently reported by Allievi et al. and Donas et al. [17, 18] by the use of the new Shockwave L6 IVL 12 mm catheter to treat extensive infra-paravisceral aorta calcifications, confirming that lithotripsy can be considered as a standalone therapy for CRA.

In the present report, a stentless aortic procedure was performed to treat a case of paravisceral CRA as an alternative to open surgery. Although our final angiogram revealed a suboptimal result with an apparent residual stenosis, the calcific plaque remodeling with preservation of side branches was evident, and a postprocedural improvement of hemodynamic data was achieved (Figures 3, 4). We considered the procedure to be effective without stent-stent graft implantation.

According to other studies [9, 10, 13-15], our experience suggests that IVL may be able to achieve good functional results in the treatment of peripheral obstructive disease, restoring vessel wall distensibility through fracture and redistribution of calcium, even with no stent placement. In the case of CRA, IVL can be proposed as a standalone procedure without consequences for any subsequent open surgery, still providing significant hemodynamic results [16-18]. However, the promising results of endovascular lithotripsy should be considered as preliminary, and further clinical experience is required to define the long-term role of this procedure.

## Conclusions

While CRA is usually treated by open surgery, the use of novel techniques such as IVL balloon angioplasty may be considered as an effective therapy associated with low morbidity. This technique has the potential to remold the aortic calcification, improving blood flow without the strict need for stenting. The patient was a candidate for several treatment options, including double-barrel stenting and open surgery, but we preferred to perform IVL, considering the low risk of the procedure and patient comfort. One month later, our patient is symptom-free, showing no sign of restenosis at vascular ultrasound assessment. A three-month follow-up has been planned to evaluate the anatomoclinical evolution.

IVL is a safe and effective option for patients suffering from arterial disease with extensive calcifications. In the case of CRA, the promising results of endovascular lithotripsy should be considered as preliminary, and further clinical experience is required to define the long-term role of this procedure.
